# Perceived Mistreatment and Professional Identity of Medical Students in China

**DOI:** 10.1001/jamanetworkopen.2024.44245

**Published:** 2024-11-08

**Authors:** Xuanxuan Ma, Ziyue Shen, Ruilian Xiao, Hongbin Wu

**Affiliations:** 1 School of Public Health, Peking University, Haidian District, Beijing, China; 2 Graduate School of Education, Peking University, Haidian District, Beijing, China; 3 Institute of Medical Education, Peking University, Haidian District, Beijing, China

## Abstract

**Importance:**

Mistreatment is a common experience among medical students, with various negative consequences of such perceived mistreatment reported. However, few large-scale studies have investigated the association between perceived mistreatment and the formation of medical students’ professional identities.

**Objective:**

To investigate medical students’ perceived mistreatment during medical school and its association with professional identity.

**Design, Setting, and Participants:**

This repeated cross-sectional study included medical students graduating between 2019 and 2022 at 135 medical schools in China. Analyses were performed from July 25, 2023, to May 15, 2024.

**Exposure:**

The China Medical Student Survey includes 5 items designed to measure students’ perceived experiences of mistreatment.

**Main Outcomes and Measures:**

Professional identity was measured using a validated 7-item scale adapted from the Macleod Clark Professional Identity Scale. Multivariate linear regression was used to determine the association between perceived mistreatment and medical students’ professional identity.

**Results:**

A total of 94 153 students (53 819 female [57.2%]; 83 548 Han ethnicity [88.7%]) were analyzed, representing 67.2% of the medical graduates enrolled in all responding medical schools. Most medical students reported having experienced at least 1 mistreatment incident (79 554 students [84.5%]). Medical students reported being required to perform personal service (57 455 students [61.0%]), experiencing mistreatment by patients (67 439 students [71.6%]), being publicly humiliated (24 348 students [25.9%]), being unjustly treated (35 926 students [38.2%]), and experiencing deliberate harassment (46 082 students [48.9%]). A negative association and saturation effect (where effect size plateaus after moderate exposure of mistreatment) were found between the degree of mistreatment and medical students’ professional identity scores. Compared with students who had not reported mistreatment, students who reported single (β, −0.30; 95% CI, −0.33 to −0.28; *P* < .001), moderate (β, −0.66; 95% CI, −0.69 to −0.63; *P* < .001), and high (β, −0.62; 95% CI, −0.65 to −0.58; *P* < .001) frequency of mistreatment were more likely to have lower professional identity scores; this association persisted but was attenuated after adjusting for students’ sociodemographic characteristics and was consistent across all the types of mistreatment.

**Conclusions and Relevance:**

In this national, repeated cross-sectional study, a high prevalence of mistreatment among medical students in China and a negative association between perceived mistreatment and medical students’ professional identity was found. Further research is needed to ensure that medical schools offer supportive and respectful learning environments.

## Key Points

**Question:**

Is perceived mistreatment during medical school associated with medical students’ professional identity?

**Findings:**

In this cross-sectional study of 94 153 graduating medical students from 4 cohorts in China, 84.5% reported at least 1 incident of mistreatment. A significant negative correlation was observed between perceived mistreatment and professional identity.

**Meaning:**

These findings suggest that efforts to address medical students’ mistreatment are needed to mitigate the adverse consequences on their professional identity.

## Teaser

This cross-sectional study examines the association of experiences of mistreatment during medical school with professional identity among graduating medical students in China.

## Introduction

Mistreatment is a common and damaging experience during medical school worldwide.^1^ Despite increased awareness of mistreatment and efforts to foster a supportive learning environment, prevalence of perceived mistreatment persists.^2^ Numerous studies have shown its adverse effect on medical students’ well-being,^3,4^ career satisfaction,^5^ and professional development,^5,6^ which, in turn, are associated with quality of care and attrition from the medical profession.^7,8^

Of particular concern is the association between perceived mistreatment and medical students’ professional identity, defined as “a representation of self, achieved in stages over time during which the characteristics, values, and norms of the medical profession are internalized.”^9,10^ Professional identity plays an important role in medical education and was highlighted in the 2010 Carnegie Foundation Report.^11^ The formation of professional identity is a complex and dynamic process influenced by educational experiences, role models or mentors, learning environment, and treatment by individuals in the learning environment.^9^ From the theoretical lens of social learning theory, mistreatment in the clinical learning environment might have a considerable negative impact on medical students’ professional identity, as professional identity is acquired through interaction with instructors and peers, patients, and other members of the health care system.^12,13^ Positive social interactions facilitate the internalization of medical professional cultural values and guide medical students to “think, act, and feel like physicians.”^10,14^ However, experiences of mistreatment may lead to negative perceptions of the medical profession and present challenges to the formation of their professional identity, with negative consequences.^15^ A dose-response association between the degree of mistreatment and the risk of burnout has been found in surveys of medical students.^5,16^ However, there is limited understanding of the association between perceived mistreatment and medical students’ professional identity. Moreover, most relevant studies have been conducted among physicians and medical students in certain high income countries,^16,17^ whereas to our knowledge the prevalence of mistreatment and its negative effects among Chinese medical students have not been documented.

To address this gap, this study examined the prevalence of perceived mistreatment among 4 national contemporary cohorts of graduating medical students in China and evaluated the association between the degree of perceived mistreatment and medical students’ professional identity. This study’s findings can help inform efforts to develop medical students’ professional identity more effectively, which is a core objective of medical education.

## Methods

### Data Source and Study Sample

This study used data from the China Medical Student Survey (CMSS), an annual national survey conducted by the National Center for Health Professions Education Development (NCHPED) since 2019. The CMSS aims to collect self-reported data on the demographic characteristics and learning experiences of medical students. Medical students complete the CMSS shortly before the end of each academic year, typically from May to June. The completion and return of the self-administered questionnaire were considered as informed consent. The Peking University institutional review board approved the survey. We conducted the study analyses according to the Strengthening the Reporting of Observational Studies in Epidemiology (STROBE) reporting guideline for cross-sectional data.

This study analyzes a sample of graduates of a 5-year medical program, which is a typical duration for undergraduate medical education in China. The sample includes graduates from 4 consecutive cohorts, specifically those who graduated between 2019 and 2022. These graduates, who completed their 5 years of medical training and took the CMSS survey shortly before graduation, came from 135 medical schools (32, 104, 117, and 99 schools, respectively) over the 4 years. See eAppendices 1 through 3 in Supplement 1 for additional details regarding the context of medical education in China, the CMSS, and the selected survey instrument.

### Mistreatment Measurements

The CMSS includes 5 items designed to measure students’ perceived experiences of mistreatment: being required to perform personal services by persons in positions of higher authority, mistreatment by patients, public humiliation, unjust treatment, and deliberate harassment. Responses are assessed using a 5-point scale (representing “never,” “once,” “occasionally,” “often,” and “frequently”), with answers reflecting the frequency, not the severity, of perceived mistreatment. Given the possibility that respondents could not consistently distinguish between often and frequently, they were combined to create a 4-point scale (never, single, moderate, and high). By combining the single, moderate, and high categories, we also created a dichotomous “never vs at least once” variable to determine whether the students reported ever having experienced mistreatment.

To quantify mistreatment, a summary variable was created to reflect 4 varying degrees of mistreatment: never (no mistreatment experienced), single (1 or more types of mistreatment experienced once), moderate (1 or more types of mistreatment experienced occasionally), and high (any type of mistreatment experienced frequently).^16,18^

### Professional Identity

A 7-item scale was designed to measure the professional identity of medical students based on the Macleod Clark Professional Identity Scale (MCPIS) (example item, “I am proud to be a medical student when I interact with other students”).^19^ Responses consisted of options on a 5-point Likert-type scale ranging from 1 (“strongly disagree”) to 5 (“strongly agree”). The professional identity sum score was calculated and ranged from 7 to 35. Cronbach α was calculated to determine the internal consistency of the structural and functional parameters of the professional identity scale. For each year, the Cronbach α values were larger than the usually acceptable threshold value of 0.70 (Cronbach α from 2019 to 2022 was 0.87, 0.90, 0.89, and 0.91, respectively).^20^ Exploratory factor analyses were then conducted to establish construct validity. The factor loadings of the 7-item scale ranged between 0.71 and 0.85, with an eigenvalue greater than 1 explaining a total mean variance of 62.8% (total variance from 2019 to 2022 was 57.4%, 63.6%, 61.2%, and 67.0%, respectively), which provides validity evidence for the scale.^21^ Factor scores were calculated for each participant from the 4 cohorts based on the factor loadings, and these scores were used as dependent variables.

### Explanatory Covariates

The covariates included sex, ethnicity (Han Chinese , other ethnicity), hometown (urban, rural), annual household income (low, middle, high), National College Entrance Examination (NCEE) score, financial pressure (yes, no), and grade point average (GPA) ranking (top 25%, bottom 75%). Financial pressure was investigated by asking the participants whether they felt financial pressure. NCEE scores and GPA rankings indicated academic achievement at enrollment and their self-assessment in the current year, respectively.

### Statistical Analyses

We used χ^2^ test and Kruskal-Wallis tests, as appropriate, to compare differences in the characteristics, professional identity scores, and perceived mistreatment of participants by cohort.

As medical students were nested within medical schools, we successively employed an unconditional multilevel model, commonly referred to as the null model, to estimate the intraclass correlation coefficient (ICC) to identify the proportion of variance in the *z*-score professional identity attributed to clustering between schools.^22^ Preliminary analyses indicated that the variance in professional identity scores attributable to schools was minimal (ICC from 2019 to 2022: 5.6%, 3.9%, 3.4%, and 4.4%, respectively), allowing us to treat students’ professional identity scores as independent. Multivariable linear regression was then used to determine the association between degrees of perceived mistreatment, as well as the frequencies of different types of mistreatment, and medical students’ professional identity scores after adjusting for student demographic characteristics. Although preliminary analyses suggested that professional identity scores could be regarded as independent, we controlled for school fixed effects and computed 95% CIs using robust standard errors. Collinearity among covariates was assessed by examining variance inflation factors; no evidence of collinearity was found.

Statistical analyses were performed from July 25, 2023, to May 15, 2024, using R version 4.3.2 (R Project for Statistical Computing). All tests were 2-tailed, and *P* < .05 was considered statistically significant.

## Results

The initial study sample included 97 244 students. Of these, 1775 students (1.8%) were excluded because they did not respond to all mistreatment questions and 1316 (1.4%) were excluded because they did not respond to the professional identity scale. Therefore, our analysis included 94 153 students (53 819 female [57.2%]), representing approximately 67.2% of the medical graduates enrolled in all responding medical schools at the time of data collection (Table 1). From 2019 to 2022, there were 7260, 27 775, 33 958, and 25 160 respondents, respectively. Most respondents were Han Chinese (83 548 students [88.7%]); 50 317 students (53.4%) were from urban areas; 40 317 students (42.8%) were from low-income, 44 306 (47.1%) from middle-income, and 9530 (10.1%) from high-income families, respectively; 59 714 (63.4%) reported financial pressure, and 33 681 (35.8%) reported GPAs in the top 25%. The median (IQR) professional identity total score was 27.0 (24.0-30.0). More details regarding the professional identity scores are given in eTable 1 in Supplement 1.

**Table 1.** Characteristics of Respondents to the CMSS Questionnaire, 2019-2022

Characteristic	Respondents, No. (%)^a^					*P* value^b^
	Total (n = 94 153)	2019 (n = 7260)	2020 (n = 27 775)	2021 (n = 33 958)	2022 (n = 25 160)	
Sex						
Female	53 819 (57.2)	4017 (55.3)	16 543 (59.6)	19 074 (56.2)	14 185 (56.4)	<.001
Male	40 334 (42.8)	3243 (44.7)	11 232 (40.4)	14 884 (43.8)	10 975 (43.6)	
Ethnicity						
Han Chinese	83 548 (88.7)	6343 (87.4)	24 339 (87.6)	30 367 (89.4)	22 499 (89.4)	<.001
Other ethnicity	10 605 (11.3)	917 (12.6)	3436 (12.4)	3591 (10.6)	2661 (10.6)	
Hometown						
Urban	50 317 (53.4)	4542 (62.6)	14 010 (50.4)	18 028 (53.1)	13 737 (54.6)	<.001
Rural	43 836 (46.6)	2718 (37.4)	13 765 (49.6)	15 930 (46.9)	11 423 (45.4)	
Annual household income^c^						
Low	40 317 (42.8)	2571 (35.4)	13 505 (48.6)	13 939 (41.0)	10 302 (40.9)	<.001
Middle	44 306 (47.1)	3894 (53.6)	12 894 (46.4)	15 787 (46.5)	11 731 (46.6)	
High	9530 (10.1)	795 (11.0)	1376 (5.0)	4232 (12.5)	3127 (12.4)	
NCEE score						
Mean (SD)	537.6 (59.8)	566.2 (76.7)	534.6 (63.1)	543.0 (55.0)	525.2 (52.6)	NA
Median (IQR)	540.0 (500.0-575.0)	565.7 (536.0-617.0)	535.0 (495.0-579.0)	544.4 (512.0-577.0)	530.0 (487.0-568.0)	<.001
Financial pressure						
No	34 439 (36.6)	3703 (51.0)	9195 (33.1)	12 061 (35.5)	9480 (37.7)	<.001
Yes	59 714 (63.4)	3557 (49.0)	18 580 (66.9)	21 897 (64.5)	15 680 (62.3)	
GPA rank						
Top 25%	33 681 (35.8)	2329 (32.1)	11 347 (40.9)	10 544 (31.1)	9461 (37.6)	<.001
Bottom 75%	60 472 (64.2)	4931 (67.9)	16 428 (59.1)	23 414 (68.9)	15 699 (62.4)	
Professional identity score						
Mean (SD)	27.0 (4.8)	27.2 (5.4)	27.3 (4.7)	26.7 (4.6)	27.0 (4.9)	NA
Median (IQR)	27.0 (24.0-30.0)	28.0 (23.0-32.0)	28.0 (24.0-30.0)	27.0 (23.0-29.0)	27.0 (23.0-30.0)	<.001

Abbreviations: CMSS, China Medical Student Survey; GPA, grade point average; NA, not applicable; NCEE, National College Entrance Examination.

^a^ Percentages have been rounded and may not total 100.

^b^ Comparison among 4 groups was assessed by analysis of the χ^2^ test (for categorical variables) and Kruskal-Wallis (for NCEE score and professional identity score) test as appropriate.

^c^ In this study, annual household income is grouped into 3 categories based on the context in China. Incomes below 10 000 renminbi (RMB) and between 10 000 RMB and 30 000 RMB are defined as low income. Incomes between 30 000 RMB and 150 000 RMB are classified as middle income, while incomes above 150 000 RMB are considered high income.

Overall, 79 554 students (84.5%) reported at least 1 incident of mistreatment, with the proportion ranging from 27 859 of 33 958 respondents (82.0%) in 2021 to 22 631 of 25 160 respondents (89.9%) in 2022 (Table 2). Concerning the degree of mistreatment, 38 482 students (40.9%) reported a single instance, 26 905 (28.6%) moderate frequency, and 14 167 (15.0%) high frequency of mistreatment, respectively. Among the 5 types of reported mistreatment, mistreatment by patients was the most common (67 439 students [71.6%]), ranging from 22 145 of 33 958 respondents (66.0%) in 2021 to 20 667 of 25 160 respondents (82.1%) in 2022; 57 455 students (61.0%) reported having been required to perform personal services, ranging from 19 815 of 33 958 respondents (58.4%) in 2021 to 4686 of 7260 respondents (64.5%) in 2019; 46 082 students (48.9%) reported deliberate harassment, ranging from 14 936 of 33 958 respondents (44.0%) in 2021 to 14 749 of 27 775 respondents (53.1%) in 2020; 35 926 students (38.2%) reported unjust treatment, ranging from 11 166 of 33 958 respondents (32.9%) in 2021 to 10 513 of 25 160 respondents (41.8%) in 2022; and 24 348 students (25.9%) reported public humiliation, ranging from 7758 of 33 958 respondents (22.8%) in 2021 to 8155 of 27 775 respondents (29.4%) in 2020.

**Table 2.** Chinese Medical Students’ Experiences of Mistreatment, 2019-2022

Variables	Respondents, No. (%)^a^					*P* value
	Total (n = 94 153)	2019 (n = 7260)	2020 (n = 27 775)	2021 (n = 33 958)	2022 (n = 25 160)	
**Required to perform personal services**						
Never	36 698 (39.0)	2574 (35.5)	10 767 (38.8)	14 143 (41.6)	9214 (36.6)	<.001^b^
Single	30 059 (31.9)	1554 (21.4)	8589 (30.9)	10 617 (31.3)	9299 (37.0)	
Moderate	17 855 (19.0)	1523 (21.0)	5541 (19.9)	6480 (19.1)	4311 (17.1)	
High	9541 (10.1)	1609 (22.2)	2878 (10.4)	2718 (8.0)	2336 (9.3)	
At least once	57 455 (61.0)	4686 (64.5)	17 008 (61.2)	19 815 (58.4)	15 946 (63.4)	<.001^c^
**Mistreatment by patients**						
Never	26 714 (28.4)	1960 (27.0)	8718 (31.4)	11 543 (34.0)	4493 (17.9)	<.001^b^
Single	41 425 (44.0)	2177 (30.0)	11 542 (41.6)	14 273 (42.0)	13 433 (53.4)	
Moderate	20 460 (21.7)	2050 (28.2)	5830 (21.0)	6842 (20.1)	5738 (22.8)	
High	5554 (5.9)	1073 (14.8)	1685 (6.1)	1300 (3.8)	1496 (5.9)	
At least once	67 439 (71.6)	5300 (73.0)	19 057 (68.6)	22 415 (66.0)	20 667 (82.1)	<.001^c^
**Public humiliation**						
Never	69 805 (74.1)	5168 (71.2)	19 620 (70.6)	26 200 (77.2)	18 817 (74.8)	<.001^b^
Single	11 724 (12.5)	915 (12.6)	3555 (12.8)	3552 (10.5)	3702 (14.7)	
Moderate	9902 (10.5)	703 (9.7)	3395 (12.2)	3655 (10.8)	2149 (8.5)	
High	2722 (2.9)	474 (6.5)	1205 (4.3)	551 (1.6)	492 (2.0)	
At least once	24 348 (25.9)	2092 (28.8)	8155 (29.4)	7758 (22.8)	6343 (25.2)	<.001^c^
**Unjust treatment**						
Never	58 227 (61.8)	4439 (61.1)	16 349 (58.9)	22 792 (67.1)	14 647 (58.2)	<.001^b^
Single	19 703 (20.9)	1266 (17.4)	5799 (20.9)	5999 (17.7)	6639 (26.4)	
Moderate	12 268 (13.0)	947 (13.0)	4062 (14.6)	4321 (12.7)	2938 (11.7)	
High	3955 (4.2)	608 (8.4)	1565 (5.6)	846 (2.5)	936 (3.7)	
At least once	35 926 (38.2)	2821 (38.9)	11 426 (41.1)	11 166 (32.9)	10 513 (41.8)	<.001^c^
**Deliberate harassment**						
Never	48 071 (51.1)	3768 (51.9)	13 026 (46.9)	19 022 (56.0)	12 255 (48.7)	<.001^b^
Single	26 824 (28.5)	1537 (21.2)	7968 (28.7)	8862 (26.1)	8457 (33.6)	
Moderate	14 363 (15.3)	1153 (15.9)	4855 (17.5)	4957 (14.6)	3398 (13.5)	
High	4895 (5.2)	802 (11.0)	1926 (6.9)	1117 (3.3)	1050 (4.2)	
At least once	46 082 (48.9)	3492 (48.1)	14 749 (53.1)	14 936 (44.0)	12 905 (51.3)	<.001^c^
**Degree of mistreatment**						
Never	14 599 (15.5)	1053 (14.5)	4918 (17.7)	6099 (18.0)	2529 (10.1)	<.001^b^
Single	38 482 (40.9)	1651 (22.7)	10 992 (39.6)	14 329 (42.2)	11 510 (45.7)	
Moderate	26 905 (28.6)	2230 (30.7)	7887 (28.4)	9402 (27.7)	7386 (29.4)	
High	14 167 (15.0)	2326 (32.0)	3978 (14.3)	4128 (12.2)	3735 (14.8)	
At least once	79 554 (84.5)	6207 (85.5)	22857 (82.3)	27 859 (82.0)	22 631 (89.9)	<.001^c^

^a^ Percentages have been rounded and may not total 100.

^b^ *P* value represents the χ^2^ test for differences in the 4-point mistreatment response across the 4 cohorts.

^c^ *P* value represents the χ^2^ test in the dichotomous never vs at least once mistreatment variable across the 4 cohorts.

**Figure 1.**
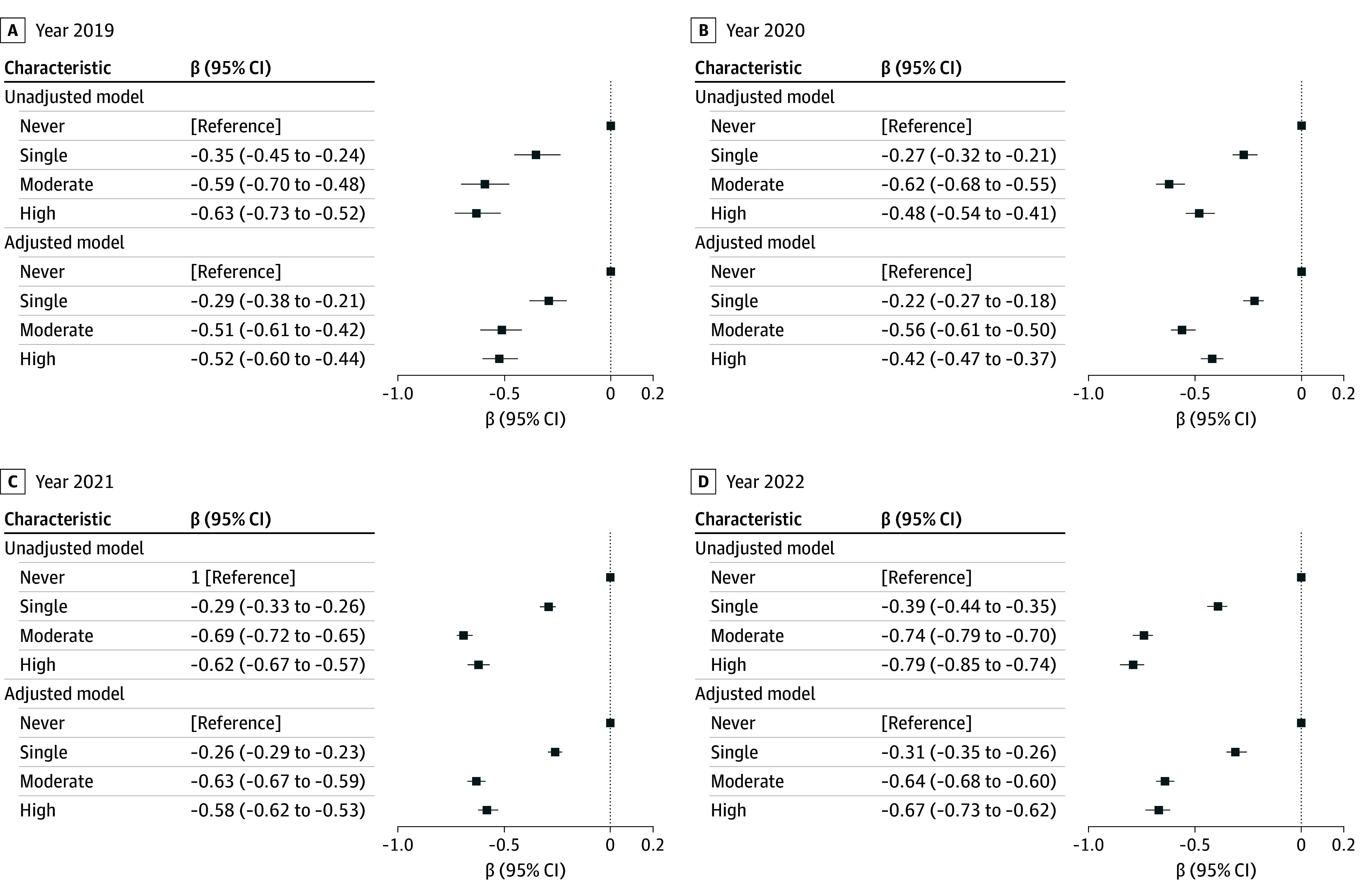
Degree of Medical Students’ Perceived Mistreatment and Professional Identity, 2019-2022

In multivariate regression analyses, there was a negative association and saturation effect (where effect size plateaus after moderate exposure of mistreatment) between the degree of perceived mistreatment and professional identity scores after adjusting for student characteristics (Figure 1; eTable 2 in Supplement 1). Compared with students who had no experiences of perceived mistreatment, students who reported a single perceived mistreatment (β, −0.25; 95% CI, −0.28 to −0.23; *P* < .001) were more likely to have a lower professional identity score, with the β coefficient ranging from −0.22 (95% CI, −0.27 to −0.18; *P* < .001) in 2020 to −0.31 (95% CI, −0.35 to −0.26; *P* < .001) in 2022. The absolute value of the β coefficient was highest for students who reported moderate perceived mistreatment (β, −0.60; 95% CI, −0.63 to −0.57; *P* < .001) compared with students who reported single and high perceived mistreatment. The absolute value of the β coefficient among students who reported high perceived mistreatment (β, −0.55; 95% CI, −0.58 to −0.52; *P* < .001) was higher than that among students who reported single perceived mistreatment, but slightly lower than those who reported moderate perceived mistreatment. This indicated a saturation effect of the association at a moderate frequency of mistreatment, a pattern predominantly observed in 2020 and 2021. However, the absolute value of the β coefficient among students who reported high perceived mistreatment was slightly higher than that of students who reported moderate perceived mistreatment in the 2019 and 2022 samples.

**Figure 2.**
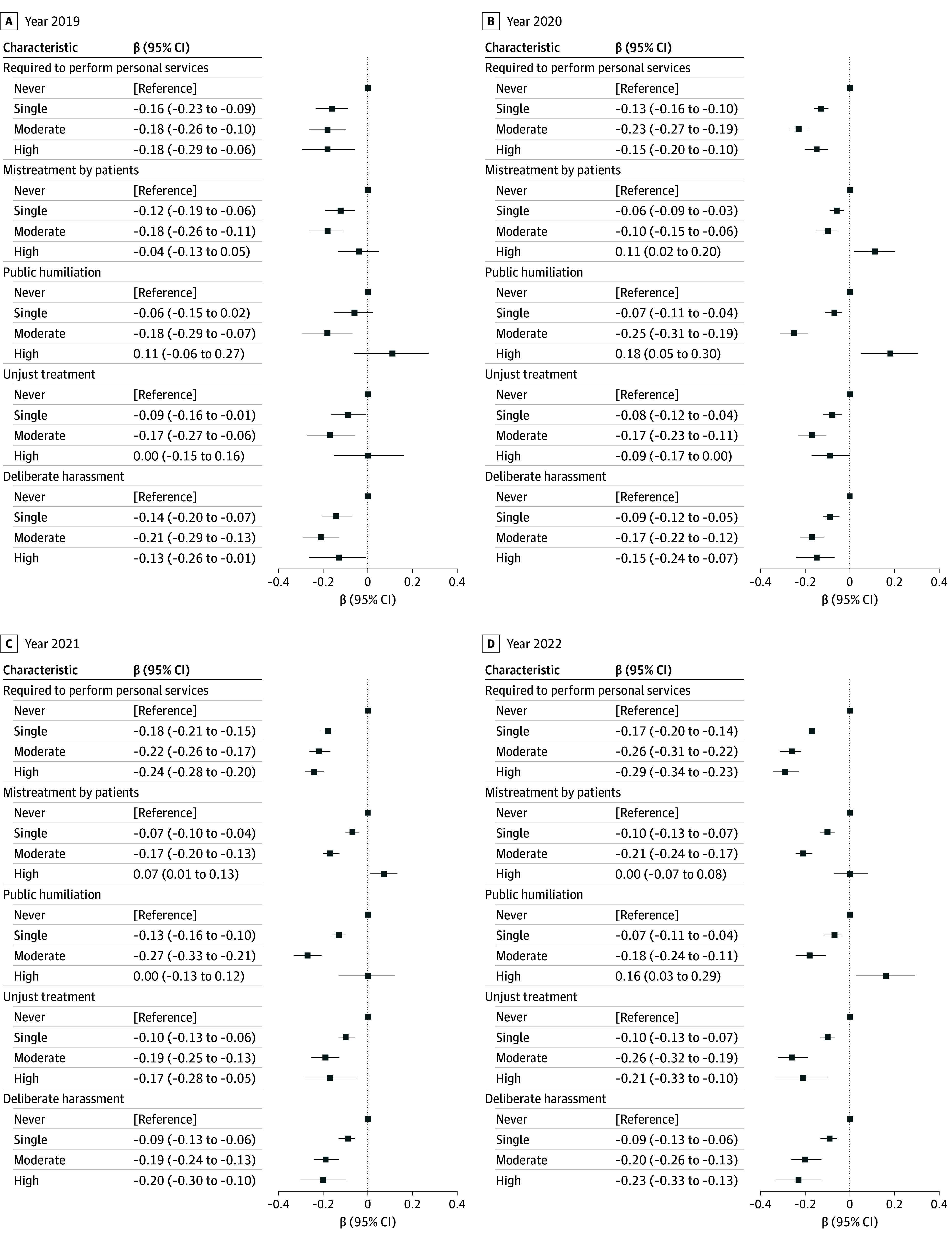
Frequencies of Each Type of Medical Students’ Mistreatment and Professional Identity, 2019-2022

We also explored the negative association and saturation effect between the frequency of the 5 types of mistreatment and medical students’ professional identity scores (Figure 2; eTable 3 in Supplement 1). After adjusting for student demographic factors, the students who reported a single incident of having been required to perform personal services (β, −0.16; 95% CI, −0.17 to −0.14; *P* < .001) had lower professional identity scores. The absolute value of the β coefficient among students who reported a moderate level of having been required to perform personal services (β, −0.23; 95% CI, −0.25 to −0.21; *P* < .001) was significantly higher compared with the coefficient of those who reported a single incident. There was no significant difference between the β coefficient of moderate and high perceived mistreatment (β, −0.22; 95% CI, −0.25 to −0.19; *P* < .001), indicating a saturation effect of the association at a moderate frequency of mistreatment. This pattern was also observed in the association between professional identity scores and the increased frequency of unjust treatment (single: β, −0.09; 95% CI, −0.11 to −0.07; *P* < .001; moderate: β, −0.21; 95% CI, −0.24 to −0.18; *P* < .001; high: β, −0.13; 95% CI, −0.19 to −0.07; *P* < .001) or of deliberate harassment (single: β, −0.09; 95% CI, −0.12 to −0.07; *P* < .001; moderate: β, −0.19; 95% CI, −0.23 to −0.16; *P* < .001; high: β, −0.18; 95% CI, −0.23 to −0.12; *P* < .001). When comparing students with no reports of mistreatment by patients, students who experienced a single incident (β, −0.07; 95% CI, −0.09 to −0.05; *P* < .001) or moderate mistreatment (β, −0.16; 95% CI, −0.18 to −0.13; *P* < .001) by patients had lower professional identity scores. However, while the β coefficient of high mistreatment (β, 0.06; 95% CI, 0.02 to 0.11; *P* < .01) was positive, it had no statistical difference in 2022 (β, 0.003; 95% CI, −0.07 to 0.08; *P* = .92). In addition, the coefficient of experiencing high frequencies of humiliation (β, 0.15; 95% CI, 0.09 to 0.22; *P* < .001) was also positive but with no statistical difference in 2019 (β, 0.11; 95% CI, −0.06 to 0.27; *P* = .19).

## Discussion

To our knowledge, this is the first comprehensive study to investigate Chinese medical graduates’ experience of mistreatment and the association between perceived mistreatment and professional identity. We identified a high prevalence of mistreatment among medical students. Adjusting for student characteristics, the degree of perceived mistreatment and medical students’ professional identity were negatively associated, with a saturation effect of the association at a moderate frequency of mistreatment. This pattern also appeared in the association between the frequency of the 5 types of mistreatment investigated and medical students’ professional identity. The results were largely consistent across the 4 cohorts of CMSS data.

The overall degree of mistreatment found in this study accords with that reported in a previous longitudinal project (84.5% vs 83%, respectively),^5^ but much higher than the Association of American Medical Colleges (AAMC) Graduation Questionnaire (GQ) 2016-2017 data (84.5% vs 46%, respectively).^18^ While these differences may be due to differences in measurement methods, our comparison of similar mistreatment items revealed that the prevalence of mistreatment in China was higher than in GQ data, as was the case for being required to perform personal services (between 58.4% and 64.5% in the CMSS data vs 3.2% in GQ 2024) and public humiliation (between 22.8% and 29.4% in CMSS vs 20.1% in GQ 2024).^23^ While these varying findings suggest apparent variability in cultures and learning environments across countries,^24,25^ it is also possible that because the average age of the graduates in this study was approximately 4 years younger than that of graduates in the US, this made them more likely to perceive and find it challenging to confront mistreatment during medical training.^16,22,26^

This study found the highest prevalence of perceived mistreatment by patients. Patients and their families are regarded as a common source of mistreatment in learning environments.^27^ Our findings accord with prior research while adding nuance to the quantitative frequencies of mistreatment by patients.^28^ Additionally, being required to complete personal tasks by individuals in higher positions was found to be significant more frequent than in GQ data.^24^ Requiring medical students to complete tasks unrelated to or beyond their learning scope is reportedly common.^29,30^ Fitting in with an established hierarchy of academic authority,^31^ medical students tend to obey instructions from superiors.^29^ Although not involving direct verbal abuse or physical harm, this represents an abuse of power wherein superiors leverage their authority over students.^32^ Medical students in China appear to bear a greater burden of power abuse during medical education. Varying levels of power distance, a criterion measuring the degree of power inequality acceptance,^33^ could also explain the results. In societies characterized by high power distance, such as China, people in higher positions are more absolute in their expectation for subordination.^29,34^ These findings may be useful in developing interventions and could be extended to future studies in other typically high–power distance societies.^35,36^

Notably, perceived mistreatment was negatively associated with medical students’ professional identity, which accords with previous findings that a negative learning environment and a culture lacking respect are detrimental to medical students’ professional identity.^37^ The coefficients of high mistreatment were not significantly larger or even smaller than moderate mistreatment, indicating a saturation effect in terms of the increasing degree of perceived mistreatment and medical students’ professional identity. A possible explanation could be psychological rationalization, used for protection from anxiety or threats to self-esteem.^38,39^ Some medical students and educators believe that experiences of mistreatment and their subsequent rationalization of those adverse events are part of their identity formation as health care professionals.^40^ Further research is needed to explore how medical students cope with mistreatment and the long-term impacts of mistreatment on the formation of their professional identity.

Our findings suggest that creating a supportive and nurturing learning environment is essential for promoting professional identity among medical students. Theoretically, students acquire the values that will be basic to their professional way of life and learn to play the role of a physician through the process of socialization within the community of practice, including observing and evaluating the behavior of their supervisors and exchanging experiences and ideas with peers, patients, and other members of the health system.^41^ Students’ encounters of mistreatment during the critical stages of professional identity formation can negatively impact their subjective well-being,^42^ retention in the education program,^19^ physicians’ professional development,^43^ and physicians’ good relationship with their patients.^8,10^ Here, we highlight some important considerations for leaders in academic medicine, national medical organizations, and medical schools when implementing effective strategies to improve interactions between students and others in the medical community. Additionally, while the highest degree of mistreatment did not inevitably result in the lowest professional identity scores, a high degree of mistreatment may still not necessarily have a less adverse impact on professional identity. Further investigation is recommended as mistreatment can sometimes be ignored and frequently rationalized as a useful means to help students become better physicians.^44^ Medical students who use rationalization might downplay not only their own adverse experiences but also other people’s similar difficulties.^39^ Therefore, students who experience mistreatment may be more likely to mistreat their future students and patients, eventually forming a vicious circle.^45^ To reduce the adverse impacts of suppressing feelings, both institutional efforts and individual-level solutions to improve their learning experiences and support professional identity development are needed. The Yale School of Medicine conducts annual Power Day workshops and provides safe spaces for advice, support, and professional identity formation.^32^ Further interventions and evaluation of these interventions are needed to address mistreatment and improve the learning environment for medical students.

This study contributes to existing research in several ways. First, this study includes data from 135 medical schools, covering a significant portion of China’s 202 medical schools,^46^ with sample sizes ranging from 7260 to 33 958 across 4 cohorts. Given that around 94 600 medical students graduate annually,^47^ this broad institutional and sample coverage maximizes the generalizability of the findings to Chinese medical schools. Second, these national survey data provide a new perspective on the topic of medical student mistreatment in the Chinese context, which can serve as a foundation for future cross-cultural comparisons. Third, the study findings add to research on the negative consequences of student mistreatment by exploring the association between perceived mistreatment and medical students’ professional identity, which to our knowledge has not been previously described. Last, this study revealed a saturation effect in the association of mistreatment with professional identity, indicating the potential presence of a rationalization mechanism employed to cope with mistreatment. However, the generalizability of this saturation effect requires further investigation.

### Limitations

This study has several limitations. First, it relied on medical students’ self-reported experiences of mistreatment during undergraduate medical education, and definitions of negative behaviors constituting mistreatment may vary among medical students; thus, the data are subject to the limitations of reporting bias. Additionally, missing data could cause selection bias. Second, the items investigating mistreatment in the CMSS were designed based on GQ data, Chinese society and culture, and expert consultations, which may only represent mistreatment in similar cultural contexts. As a repeated cross-sectional survey, this study could explore associations but could not establish causation. Moreover, professional identity is dynamic and ever-changing. Future studies should leverage longitudinal data to determine causal explanations of the underlying mechanisms that explain the negative association between perceived mistreatment and medical students’ professional identity and consider related heterogeneity.

## Conclusions

This study revealed a high prevalence of mistreatment among Chinese medical students and a negative association and saturation effect between medical students’ perceived mistreatment and their professional identity. Effective strategies are required to create supportive and respectful learning environments, involving both institutional efforts and individual-level solutions.

## Notes

Supplement 1. **eAppendix 1.** Context: Medical Education in China **eAppendix 2.** The China Medical Student Survey (CMSS) **eAppendix 3.** Survey Instrument: Selected Items From China Medical Student Survey (CMSS) **eTable 1.** Graduating Chinese Medical Student Self-Reporting Professions Identity, 2019-2022 **eTable 2.** The Association Between the Degree of Medical Students’ Perceived Mistreatment and Professional Identity, 2019-2022 **eTable 3.** The Association Between the Frequencies of Each Type of Medical Students’ Mistreatment and Professional Identity, 2019-2022

Supplement 2.
Data Sharing Statement
