# Supplementary material for: Perceived Mistreatment and Professional Identity of Medical Students in China
Source: JAMA Netw Open. 2024 Nov 8;7(11):e2444245. doi: 10.1001/jamanetworkopen.2024.44245 (PMC11549658; doi:10.1001/jamanetworkopen.2024.44245)
Supplement: Supplement 1. — eAppendix 1. Context: Medical Education in China eAppendix 2. The China Medical Student Survey (CMSS) eAppendix 3. Survey Instrument: Selected Items From China Medical Student Survey (CMSS) eTable 1. Graduating Chinese Medical Student Self-Reporting Professions Identity, 2019-2022 eTable 2. The Association Between the Degree of Medical Students’ Perceived Mistreatment and Professional Identity, 2019-2022 eTable 3. The Association Between the Frequencies of Each Type of Medical Students’ Mistreatment and Professional Identity, 2019-2022 [file jamanetwopen-e2444245-s001.pdf]

Supplemental Online Content

Ma X, Shen Z, Xiao R, Wu H. Perceived mistreatment and professional identity of medical students in China. *JAMA Netw Open.* 2024;7(11):e2444245. doi:10.1001/jamanetworkopen.2024.44245

eAppendix 1. Context: Medical Education in China

eAppendix 2. The China Medical Student Survey (CMSS)

eAppendix 3. Survey Instrument: Selected Items From China Medical Student Survey (CMSS)

eTable 1. Graduating Chinese Medical Student Self-Reporting Professions Identity, 2019-2022

eTable 2. The Association Between the Degree of Medical Students' Perceived Mistreatment and Professional Identity, 2019-2022

eTable 3. The Association Between the Frequencies of Each Type of Medical Students' Mistreatment and Professional Identity, 2019-2022

This supplemental material has been provided by the authors to give readers additional information about their work.

© 2024 Ma X et al. *JAMA Network Open.*

eAppendix 1. Context: Medical education in China

China, as a middle- to low-income country with a large and economically diverse population, has developed a vast and complex medical education system to address the shortage and unequal distribution of healthcare professionals.[1] The complexity of the medical education system is primarily reflected in two aspects: a three-tier structure of medical schools and a variety of education programs. There are 202 medical schools offering medical education, including 47 top-tier medical schools categorized as "Double First-Class" universities, 133 general undergraduate universities, and 22 independent colleges.[3] In terms of training programs, China mainly offers three types: a 5-year program (bachelor), a 5+3 program (master), and an 8-year program (MD). Among them, the 5-year is the typical medical program; the 8-year track is designed to produce future physician-scientists; and the '5 + 3' track will become the main direction of future development. As the main program, the five-year program delivers approximately 94,600 medical graduates each year. [2]

Medicine-related studies in China begin at the undergraduate level. High school graduates who want to become healthcare professionals were admitted to different medical training programs and different tiers of medical school depending on their National College Entrance Examination (NCEE) score. Regardless of training programs, medical students should spend five years completing general education, basic medical education, clinical medical education (including clerkship). After the fifth year, students enrolled in an eight-year program or a 5+3 program automatically transition into graduate study or standardized residency training without going through the application and admission process. Those enrolled in a five-year program could apply for graduate study or residency training. All medical students have to take the National Medical Licensing Examination (NMLE) in the second year of residency training, and only those who have passed the NMLE can obtain the physician qualification certificate which signifies the transition from student status to a member of one of the health professions.[4]

References

1. Hou J, Michaud C, Li Z, et al. Transformation of the education of health professionals in China: progress and challenges. Lancet. 2014 Aug 30;384(9945):819-27. doi: 10.1016/S0140-6736(14)61307-6. PMID: 25176552.
2. Wang W. Medical education in China: progress in the past 70 years and a vision for the future. *BMC Medical Education*. 2021;21(1):453. doi:10.1186/s12909-021-02875-6
3. National Center for Health Professions Education Development (NCHPED). Directory of Higher Education Institutions in China (Health Professions Education Category). https://medudata.meduc.cn/page/detail/615417e2b4d5221acea4a355. Published August, 2022. Accessed September 1, 2024.
4. Wang, X. Experiences, challenges, and prospects of National Medical Licensing Examination in China. BMC Med Educ 22, 349 (2022). https://doi.org/10.1186/s12909-022-03385-9

© 2024 Ma X et al. *JAMA Network Open.*

eAppendix 2. The China Medical Student Survey (CMSS)

The China Medical Student Survey (CMSS) is an annual national survey administered by the National Center for Health Professions Education Development (NCHPED). The CMSS was first administered in 2019 and is an important approach for medical schools to understand and improve medical students' experience of medical education from their perspectives.[1] The CMSS includes questions related to:

- Demographic characteristics
- Background before medical school
- Satisfaction with curriculum and teaching services
- Student competence and academic achievement
- Learning experiences
- Career plans

To ensure survey quality, the NCHPED consulted an advisory panel of experts in medicine or education, survey methodology, and international literature in the process of designing the survey instrument and conducted a pilot test before the instrument was used nationally in 2019.[2]

Medical students complete the CMSS shortly before the end of each academic year, typically from May to June. Participation in the CMSS survey is voluntary and the responses are anonymous. The completion and return of the self-administered questionnaire were considered as informed consent. The CMSS has been approved by the Peking University Institutional Review Board (IRB00001052-20069).

In 2019, the NCHPED conducted two-stage sampling based on geographic location and medical school reputation, selecting 33 schools and inviting them to participate in the CMSS. The leadership of all 33 medical schools voluntarily agreed to participate in the study. Since then, the NCHPED has opened applications to medical schools nationwide. More medical schools have expressed interest in joining as they recognized that the survey helps medical schools better understand students' experiences in medical education, which can improve educational management. The figure below illustrates the progress and improvements made in the CMSS since its implementation in 2019. The survey's instrument and implementation process have also been continuously refined. Now, the CMSS is the largest and most comprehensive survey of medical education in China.

Overview of The China Medical Student Survey (CMSS) Between 2019 and 2022

Characteristics	Year 2019	Year 2020	Year 2021	Year 2022
The number of medical schools	33	107	176	130
The number of participants	10,062	152,645	47.5 thousand	30 thousand
Grade of survey	Graduates (Fifth year)	First year to Fifth year	First year to Fifth year	First year to Fifth year
Programs	Medical program	Medical program	Five types of HPE programs*	All types of HPE programs*

© 2024 Ma X et al. *JAMA Network Open.*

The CMSS has implemented a series of measures to ensure quality and improve response rates:

- **Before the Survey:** In March each year, the NCHPED releases an announcement and registration link for the CMSS survey. Participating schools must appoint a program manager who receives training on data collection and quality control procedures. After completing the training, each program manager is provided with an account for the data platform (https://medudata.meduc.cn/).
- **During the Survey:** In mid-May, the NCHPED launches the survey. The program manager distributes the survey link to all students and reminds them to participate, emphasizing the importance of their participation in improving medical education. This emphasis helps motivate a strong response. When students open the survey link, they are presented with an informed consent form, which highlights that they could answer based on their personal experiences, as their responses will not affect them individually or their schools. This is intended to minimize biases, such as social desirability bias, and prevent students from tailoring their answers to align with perceived expectations. Throughout the survey period, the program manager can log into the data platform to monitor response rates and issue additional reminders as needed. Participation is voluntary, and students can exit the survey at any time.
- **After Data Collection:** After collecting the surveys, questionnaires are reviewed and those of poor quality are removed based on response time and quality. Criteria for determining poor quality include the presence of multiple logical errors, repeated identical answers, or duplicate entries identified by the same IP address.
- **Feedback:** After each year's survey, the NCHPED provides a feedback report to each participating medical school, detailing students' learning experiences and developmental progress. This feedback further motivates schools to participate in our survey and help them improve their medical education management.

References

1. Wu H, Xie A, Yu C, et al. Design and Implementation of the Survey on the Training and Development of Medical Students in China. Chin J Med Educ. 2021;41(2):5. doi:10.3760/cma.j.cn115259-20200328-00445. **(In Chinese)**

2. National Center for Health Professions Education Development (NCHPED). Research Report —The China Medical Student Survey (CMSS) 2022 (Medical Program). https://medu.bjmu.edu.cn/cms/show.action?code=publish_4028801e6bb6cf11016be526c0dc0014&siteid=100000&newsid=8a317c95801a48ab9bf73f5d023baf2b&channelid=0000000008. Published December, 2022. Accessed September 6, 2024.

© 2024 Ma X et al. *JAMA Network Open.*

eAppendix 3. Survey Instrument: Selected items from China Medical Student Survey (CMSS)

Part 1 Demographic characteristics

1. What is your sex?	
Male	Female
☐	☐

2. What is your ethnicity?	
Han Chinese	Other ethnic groups [please note in line]
☐	☐

3. What is your home location?	
Municipality/provincial capital (including Hong Kong, Macao and Taiwan)	☐
Prefecture-level city	☐
County-level city	☐
Villages	☐
Small towns	☐

Note: Rural–urban classification was relevant to the Chinese context. Small towns and villages were classified as rural; county-level cities, prefecture-level cities, provincial capital cities, and municipalities classified as urban.

4. How much RMB is your annual household income last year?	
Less than 10,000	☐
10,000 to 30,000	☐
30,000 to 80,000	☐
80,000 to 150,000	☐
150,000 to 300,000	☐
300,000 to 1,000,000	☐
More than 1,000,000	☐

Note: In this study, annual household income is grouped into three categories. Incomes less than 10,000 RMB and between 10,000 and 30,000 RMB are defined as low-income. Incomes between 30,000 and 150,000 RMB are classified as middle-income, while incomes above 150,000 RMB are considered high-income.

5. In the daily study life, do you feel financial pressure?	
Yes	No
☐__________	☐

Part 2 Pre-school background

6. Did you take the National College Entrance Examination?	
Yes [please note your original score.<br>If there is no original score, please fill in the rank score]	No
☐__________	☐

Part 3&4 Satisfaction with curriculum and Teaching services [omitted]

Part 5 Learning experiences

© 2024 Ma X et al. *JAMA Network Open.*

7. Please truthfully report the frequency of the following events that you have experienced during your clinical learning.					
	Never	Once	Occasionally	Often	Frequently
Required to perform personal services by persons in positions of higher authority	☐	☐	☐	☐	☐
Mistreatment by patients	☐	☐	☐	☐	☐
Public humiliation	☐	☐	☐	☐	☐
Unjust treatment	☐	☐	☐	☐	☐
Deliberate harassment	☐	☐	☐	☐	☐

Part 6 academic success and placement

8. What is your grade point average (GPA) in the current school year?	
Top 25%	☐
26-50%	☐
51-75%	☐
Bottom 25%	☐

In this study, self-reported GPA rank is divided into two categories: Top 25% and Bottom 75%. The Bottom 75% category combines the 26-50%, 51-75%, and Bottom 25% ranks into a single group.

9. Please evaluate the following statements according to your own real situation.					
	Strongly disagree	Partially disagree	Neutrality	Partially agree	Strongly agree
I recognize and comprehend the responsibilities associated with the role of a doctor.	☐	☐	☐	☐	☐
Medical education makes me determined to pursue a career as a doctor.	☐	☐	☐	☐	☐
The doctor is one of the best professions in contemporary society.	☐	☐	☐	☐	☐
I am committed to actively engaging in academic and practical endeavors that enhance my journey toward a medical career.	☐	☐	☐	☐	☐
I am proud to be a medical student when I interact with other students.	☐	☐	☐	☐	☐
I am committed to staying informed about the latest advancements in the field of medicine.	☐	☐	☐	☐	☐
I have a positive outlook on the future development of the healthcare profession.	☐	☐	☐	☐	☐

© 2024 Ma X et al. *JAMA Network Open.*

eTable 1. Graduating Chinese Medical Student Self-Reporting Professions Identity, 2019-2022

Variables	Respondents, No. (%) [a]				
	Total (N=94 153)	2019 (N=7 260)	2020 (N=27 775)	2021 (N=33 958)	2022 (N=25 160)
**I recognize and comprehend the responsibilities associated with the role of a doctor.**					
Strongly disagree	473 (0.5%)	33 (0.5%)	67 (0.2%)	254 (0.7%)	119 (0.5%)
Partially disagree	676 (0.7%)	100 (1.4%)	155 (0.6%)	276 (0.8%)	145 (0.6%)
Neutrality	10 947 (11.6%)	750 (10.3%)	2 805 (10.1%)	4 316 (12.7%)	3 076 (12.2%)
Partially agree	46 646 (49.5%)	2 517 (34.7%)	14 189 (51.1%)	17 085 (50.3%)	12 855 (51.1%)
Strongly agree	35 411 (37.6%)	3 860 (53.2%)	10 559 (38.0%)	12 027 (35.4%)	8 965 (35.6%)
**Medical education makes me determined to pursue a career as a doctor.**					
Strongly disagree	1 117 (1.2%)	192 (2.6%)	263 (0.9%)	403 (1.2%)	259 (1.0%)
Partially disagree	3 049 (3.2%)	346 (4.8%)	898 (3.2%)	1 120 (3.3%)	685 (2.7%)
Neutrality	21 916 (23.3%)	1 502 (20.7%)	5 894 (21.2%)	8 517 (25.1%)	6 003 (23.9%)
Partially agree	41 521 (44.1%)	2 546 (35.1%)	12 767 (46.0%)	15 211 (44.8%)	10 997 (43.7%)
Strongly agree	26 550 (28.2%)	2 674 (36.8%)	7 953 (28.6%)	8 707 (25.6%)	7 216 (28.7%)
**The doctor is one of the best professions in contemporary society.**					
Strongly disagree	5 087 (5.4%)	853 (11.7%)	1 442 (5.2%)	1 711 (5.0%)	1 081 (4.3%)
Partially disagree	11 014 (11.7%)	921 (12.7%)	3 355 (12.1%)	4 247 (12.5%)	2 491 (9.9%)
Neutrality	32 212 (34.2%)	1 898 (26.1%)	8 437 (30.4%)	12 866 (37.9%)	9 011 (35.8%)
Partially agree	29 458 (31.3%)	1 840 (25.3%)	9 311 (33.5%)	10 282 (30.3%)	8 025 (31.9%)
Strongly agree	16 382 (17.4%)	1 748 (24.1%)	5 230 (18.8%)	4 852 (14.3%)	4 552 (18.1%)
**I am committed to actively engaging in academic and practical endeavors that enhance my journey toward a medical career.**					
Strongly disagree	485 (0.5%)	87 (1.2%)	108 (0.4%)	181 (0.5%)	109 (0.4%)
Partially disagree	1 467 (1.6%)	222 (3.1%)	441 (1.6%)	433 (1.3%)	371 (1.5%)
Neutrality	19 824 (21.1%)	1 398 (19.3%)	4 722 (17.0%)	7 730 (22.8%)	5 974 (23.7%)
Partially agree	46 866 (49.8%)	2 816 (38.8%)	14 454 (52.0%)	17 241 (50.8%)	12 355 (49.1%)
Strongly agree	25 511 (27.1%)	2 737 (37.7%)	8 050 (29.0%)	8 373 (24.7%)	6 351 (25.2%)
**I am proud to be a medical student when I interact with other students.**					
Strongly disagree	1 024 (1.1%)	209 (2.9%)	265 (1.0%)	293 (0.9%)	257 (1.0%)
Partially disagree	3 074 (3.3%)	360 (5.0%)	990 (3.6%)	961 (2.8%)	763 (3.0%)
Neutrality	24 467 (26.0%)	1 575 (21.7%)	6 189 (22.3%)	9 637 (28.4%)	7066 (28.1%)
Partially agree	41 100 (43.7%)	2 514 (34.6%)	12 675 (45.6%)	15 006 (44.2%)	10 905 (43.3%)
Strongly agree	24 488 (26.0%)	2 602 (35.8%)	7 656 (27.6%)	8 061 (23.7%)	6169 (24.5%)

(continued)

© 2024 Ma X et al. *JAMA Network Open.*

eTable 1. Graduating Chinese Medical Student Self-Reporting Professions Identity, 2019-2022 (continued)

Variables	Respondents, No. (%) [a] Total (N=94 153)	2019 (N=7 260)	2020 (N=27 775)	2021 (N=33 958)	2022 (N=25 160)
**I am committed to staying informed about the latest advancements in the field of medicine.**					
Strongly disagree	505 (0.5%)	93 (1.3%)	134 (0.5%)	152 (0.4%)	126 (0.5%)
Partially disagree	1 997 (2.1%)	276 (3.8%)	533 (1.9%)	526 (1.5%)	662 (2.6%)
Neutrality	23 739 (25.2%)	1 631 (22.5%)	5 635 (20.3%)	8 738 (25.7%)	7 735 (30.7%)
Partially agree	44 844 (47.6%)	2 775 (38.2%)	14 101 (50.8%)	16 670 (49.1%)	11 298 (44.9%)
Strongly agree	23 068 (24.5%)	2 485 (34.2%)	7 372 (26.5%)	7 872 (23.2%)	5 339 (21.2%)
**I have a positive outlook on the future development of the healthcare profession.**					
Strongly disagree	2 791 (3.0%)	626 (8.6%)	844 (3.0%)	818 (2.4%)	503 (2.0%)
Partially disagree	7 965 (8.5%)	868 (12.0%)	2 434 (8.8%)	3 190 (9.4%)	1 473 (5.9%)
Neutrality	33 415 (35.5%)	2 153 (29.7%)	9 097 (32.8%)	13 747 (40.5%)	8 418 (33.5%)
Partially agree	34 308 (36.4%)	2 063 (28.4%)	10 601 (38.2%)	11 904 (35.1%)	9 740 (38.7%)
Strongly agree	15 674 (16.6%)	1 550 (21.3%)	4 799 (17.3%)	4 299 (12.7%)	5 026 (20.0%)

[a] Percentages have been rounded and may not total 100.

© 2024 Ma X et al. *JAMA Network Open.*

eTable 2. The Association Between the Degree of Medical Students' Perceived Mistreatment and Professional Identity, 2019-2022 [a]

Variables	Professional identity score [b]				
	Total	Year 2019	Year 2020	Year 2021	Year 2022
**Unadjusted model**					
**The degree of mistreatment**					
Never	0 [reference]	0 [reference]	0 [reference]	0 [reference]	0 [reference]
Single	-0.30 [-0.33 to -0.28] ***	-0.35 [-0.45 to -0.24] ***	-0.27 [-0.32 to -0.21] ***	-0.29 [-0.33 to -0.26] ***	-0.39 [-0.44 to -0.35] ***
Moderate	-0.66 [-0.69 to -0.63] ***	-0.59 [-0.70 to -0.48] ***	-0.62 [-0.68 to -0.55] ***	-0.69 [-0.72 to -0.65] ***	-0.74 [-0.79 to -0.70] ***
High	-0.62 [-0.65 to -0.58] ***	-0.63 [-0.73 to -0.52] ***	-0.48 [-0.54 to -0.41] ***	-0.62 [-0.67 to -0.57] ***	-0.79 [-0.85 to -0.74] ***
**Adjusted model**					
**The degree of mistreatment**					
Never	0 [reference]	0 [reference]	0 [reference]	0 [reference]	0 [reference]
Single	-0.25 [-0.28 to -0.23] ***	-0.29 [-0.38 to -0.21] ***	-0.22 [-0.27 to -0.18] ***	-0.26 [-0.29 to -0.23] ***	-0.31 [-0.35 to -0.26] ***
Moderate	-0.60 [-0.63 to -0.57] ***	-0.51 [-0.61 to -0.42] ***	-0.56 [-0.61 to -0.50] ***	-0.63 [-0.67 to -0.59] ***	-0.64 [-0.68 to -0.60] ***
High	-0.55 [-0.58 to -0.52] ***	-0.52 [-0.60 to -0.44] ***	-0.42 [-0.47 to -0.37] ***	-0.58 [-0.62 to -0.53] ***	-0.67 [-0.73 to -0.62] ***
**Sex**					
Female	0 [reference]	0 [reference]	0 [reference]	0 [reference]	0 [reference]
Male	0.05 [0.03 to 0.07] ***	0.07 [0.01 to 0.13] *	0.05 [0.02 to 0.08] **	0.04 [0.01 to 0.06] **	0.06 [0.03 to 0.09] ***
**Ethnicity**					
Other ethnicity	0 [reference]	0 [reference]	0 [reference]	0 [reference]	0 [reference]
Han Chinese	-0.04 [-0.08 to 0.00]	-0.05 [-0.15 to 0.05]	-0.03 [-0.09 to 0.04]	-0.05 [-0.10 to 0.01]	-0.04 [-0.08 to 0.01]

(continued)

© 2024 Ma X et al. *JAMA Network Open.*

eTable 2. The Association Between the Degree of Medical Students' Perceived Mistreatment and Professional Identity, 2019-2022 (continued) a

Variables	Professional identity score [b]				
	Total	Year 2019	Year 2020	Year 2021	Year 2022
**Hometown**					
Rural	0 [reference]	0 [reference]	0 [reference]	0 [reference]	0 [reference]
Urban	-0.01 [-0.02 to 0.01]	-0.03 [-0.07 to 0.01]	-0.02 [-0.05 to 0.00]	-0.01 [-0.03 to 0.02]	0.02 [-0.01 to 0.04]
**Annual household income**					
Low	0 [reference]	0 [reference]	0 [reference]	0 [reference]	0 [reference]
Median	0.01 [-0.01 to 0.02]	-0.03 [-0.09 to 0.03]	0.02 [-0.01 to 0.05]	0.02 [-0.00 to 0.04]	0.00 [-0.02 to 0.03]
High	0.05 [0.02 to 0.08] ***	-0.04 [-0.15 to 0.07]	0.09 [0.03 to 0.16] **	0.05 [0.02 to 0.09] **	0.05 [0.00 to 0.11] *
NCEE score [b]	-0.02 [-0.04 to -0.01] ***	-0.02 [-0.06 to 0.01]	-0.02 [-0.04 to 0.00]	-0.03 [-0.05 to -0.01] **	-0.03 [-0.06 to -0.01] *
**Financial Pressure**					
No	0 [reference]	0 [reference]	0 [reference]	0 [reference]	0 [reference]
Yes	-0.11 [-0.12 to -0.09] ***	-0.16 [-0.22 to -0.10] ***	-0.08 [-0.11 to -0.05] ***	-0.11 [-0.14 to -0.09] ***	-0.11 [-0.14 to -0.09] ***
**GPA rank**					
Bottom 75%	0 [reference]	0 [reference]	0 [reference]	0 [reference]	0 [reference]
Top 25%	0.17 [0.15 to 0.19] ***	0.18 [0.11 to 0.24] ***	0.15 [0.12 to 0.17] ***	0.15 [0.12 to 0.17] ***	0.21 [0.19 to 0.23] ***
**Year**					
2019	0 [reference]	NA	NA	NA	NA
2020	-0.06 [-0.13 to 0.00]	NA	NA	NA	NA
2021	-0.05 [-0.11 to 0.01]	NA	NA	NA	NA
2022	-0.02 [-0.09 to 0.04]	NA	NA	NA	NA

[a] The school fix effect was also included in the model; CI are clustered at the medical school level; * P<0.05, ** P<0.01, *** P<0.001.
[b] Standardized z scores of PI score and NCEE score were calculated and used in the regression.

© 2024 Ma X et al. *JAMA Network Open.*

eTable 3. The Association Between the Frequencies of Each Type of Medical Students' Mistreatment and Professional Identity, 2019-2022 [a]

Variables	Professional identity score [b]				
	Total	Year 2019	Year 2020	Year 2021	Year 2022
**Required to perform personal services**					
Never	0 [reference]	0 [reference]	0 [reference]	0 [reference]	0 [reference]
Single	-0.16 [-0.17 to -0.14] ***	-0.16 [-0.23 to -0.09] ***	-0.13 [-0.16 to -0.10] ***	-0.18 [-0.21 to -0.15] ***	-0.17 [-0.20 to -0.14] ***
Moderate	-0.23 [-0.25 to -0.21] ***	-0.18 [-0.26 to -0.10] ***	-0.23 [-0.27 to -0.19] ***	-0.22 [-0.26 to -0.17] ***	-0.26 [-0.31 to -0.22] ***
High	-0.22 [-0.25 to -0.19] ***	-0.18 [-0.29 to -0.06] **	-0.15 [-0.20 to -0.10] ***	-0.24 [-0.28 to -0.20] ***	-0.29 [-0.34 to -0.23] ***
**Mistreatment by patients**					
Never	0 [reference]	0 [reference]	0 [reference]	0 [reference]	0 [reference]
Single	-0.07 [-0.09 to -0.05] ***	-0.12 [-0.19 to -0.06] ***	-0.06 [-0.09 to -0.03] ***	-0.07 [-0.10 to -0.04] ***	-0.10 [-0.13 to -0.07] ***
Moderate	-0.16 [-0.18 to -0.13] ***	-0.18 [-0.26 to -0.11] ***	-0.10 [-0.15 to -0.06] ***	-0.17 [-0.20 to -0.13] ***	-0.21 [-0.24 to -0.17] ***
High	0.06 [0.02 to 0.11] **	-0.04 [-0.13 to 0.05]	0.11 [0.02 to 0.20] *	0.07 [0.01 to 0.13] *	0.00 [-0.07 to 0.08]
**Public humiliation**					
Never	0 [reference]	0 [reference]	0 [reference]	0 [reference]	0 [reference]
Single	-0.09 [-0.11 to -0.07] ***	-0.06 [-0.15 to 0.02]	-0.07 [-0.11 to -0.04] ***	-0.13 [-0.16 to -0.10] ***	-0.07 [-0.11 to -0.04] ***
Moderate	-0.23 [-0.26 to -0.19] ***	-0.18 [-0.29 to -0.07] **	-0.25 [-0.31 to -0.19] ***	-0.27 [-0.33 to -0.21] ***	-0.18 [-0.24 to -0.11] ***
High	0.15 [0.09 to 0.22] ***	0.11 [-0.06 to 0.27]	0.18 [0.05 to 0.30] **	-0.00 [-0.13 to 0.12]	0.16 [0.03 to 0.29] *
**Unjust treatment**					
Never	0 [reference]	0 [reference]	0 [reference]	0 [reference]	0 [reference]
Single	-0.09 [-0.11 to -0.07] ***	-0.09 [-0.16 to -0.01] *	-0.08 [-0.12 to -0.04] ***	-0.10 [-0.13 to -0.06] ***	-0.10 [-0.13 to -0.07] ***
Moderate	-0.21 [-0.24 to -0.18] ***	-0.17 [-0.27 to -0.06] **	-0.17 [-0.23 to -0.11] ***	-0.19 [-0.25 to -0.13] ***	-0.26 [-0.32 to -0.19] ***
High	-0.13 [-0.19 to -0.07] ***	0.00 [-0.15 to 0.16]	-0.09 [-0.17 to -0.00] *	-0.17 [-0.28 to -0.05] **	-0.21 [-0.33 to -0.10] ***

(continued)

eTable 3. The Association Between the Frequencies of Each Type of Medical Students' Mistreatment and Professional Identity, 2019-2022 (continued) [a]

Variables	Professional identity score [b]				
	Total	Year 2019	Year 2020	Year 2021	Year 2022
**Deliberate harassment**					
Never	0 [reference]	0 [reference]	0 [reference]	0 [reference]	0 [reference]
Single	-0.09 [-0.12 to -0.07] ***	-0.14 [-0.20 to -0.07] ***	-0.09 [-0.12 to -0.05] ***	-0.09 [-0.13 to -0.06] ***	-0.09 [-0.13 to -0.06] ***
Moderate	-0.19 [-0.23 to -0.16] ***	-0.21 [-0.29 to -0.13] ***	-0.17 [-0.22 to -0.12] ***	-0.19 [-0.24 to -0.13] ***	-0.20 [-0.26 to -0.13] ***
High	-0.18 [-0.23 to -0.12] ***	-0.13 [-0.26 to -0.01] *	-0.15 [-0.24 to -0.07] ***	-0.20 [-0.30 to -0.10] ***	-0.23 [-0.33 to -0.13] ***
**Sex**					
Female	0 [reference]	0 [reference]	0 [reference]	0 [reference]	0 [reference]
Male	0.07 [0.05 to 0.08] ***	0.08 [0.03 to 0.13] **	0.07 [0.03 to 0.10] ***	0.06 [0.04 to 0.09] ***	0.07 [0.04 to 0.10] ***
**Ethnicity**					
Other ethnicity	0 [reference]	0 [reference]	0 [reference]	0 [reference]	0 [reference]
Han Chinese	-0.04 [-0.08 to 0.00]	-0.04 [-0.13 to 0.06]	-0.03 [-0.09 to 0.03]	-0.06 [-0.11 to -0.00] *	-0.02 [-0.07 to 0.02]
**Hometown**					
Rural	0 [reference]	0 [reference]	0 [reference]	0 [reference]	0 [reference]
Urban	-0.00 [-0.02 to 0.01]	-0.03 [-0.06 to 0.01]	-0.02 [-0.04 to 0.00]	0.00 [-0.02 to 0.03]	0.02 [-0.00 to 0.04]
**Annual household income**					
Low	0 [reference]	0 [reference]	0 [reference]	0 [reference]	0 [reference]
Median	0.00 [-0.01 to 0.02]	-0.02 [-0.09 to 0.04]	0.01 [-0.01 to 0.04]	0.01 [-0.01 to 0.03]	-0.01 [-0.03 to 0.01]
High	0.04 [0.02 to 0.07] **	-0.03 [-0.14 to 0.08]	0.08 [0.02 to 0.15] **	0.05 [0.01 to 0.09] *	0.04 [-0.01 to 0.09]
**NCEE score [b]**	-0.02 [-0.03 to -0.01] ***	-0.03 [-0.06 to 0.01]	-0.02 [-0.04 to 0.00]	-0.03 [-0.05 to -0.01] *	-0.03 [-0.06 to -0.01] *

(continued)

© 2024 Ma X et al. *JAMA Network Open.*

eTable 3. The Association Between the Frequencies of Each Type of Medical Students' Mistreatment and Professional Identity, 2019-2022 (continued) [a]

Variables	Professional identity score [b]				
	Total	Year 2019	Year 2020	Year 2021	Year 2022
**Financial Pressure**					
No	0 [reference]	0 [reference]	0 [reference]	0 [reference]	0 [reference]
Yes	-0.09 [-0.11 to -0.08] ***	-0.14 [-0.20 to -0.08] ***	-0.07 [-0.10 to -0.04] ***	-0.10 [-0.12 to -0.08] ***	-0.09 [-0.12 to -0.06] ***
**GPA rank**					
Bottom 75%	0 [reference]	0 [reference]	0 [reference]	0 [reference]	0 [reference]
Top 25%	0.17 [0.16 to 0.19] ***	0.17 [0.11 to 0.23] ***	0.15 [0.13 to 0.17] ***	0.15 [0.12 to 0.17] ***	0.21 [0.19 to 0.23] ***
**Year**					
2019	0 [reference]	NA	NA	NA	NA
2020	0.00 [-0.06 to 0.06]	NA	NA	NA	NA
2021	-0.01 [-0.06 to 0.05]	NA	NA	NA	NA
2022	0.02 [-0.05 to 0.08]	NA	NA	NA	NA

[a] The school fix effect was also included in the model; CI are clustered at the medical school level; * P<0.05, ** P<0.01, *** P<0.001.
[b] Standardized z scores of PI score and NCEE score were calculated and used in the regression.

© 2024 Ma X et al. *JAMA Network Open.*
