# Supplementary material for: Perceived Mistreatment and Professional Identity of Medical Students in China
Source: JAMA Netw Open. 2024 Nov 8;7(11):e2444245. doi: 10.1001/jamanetworkopen.2024.44245 (PMC11549658; doi:10.1001/jamanetworkopen.2024.44245)
Supplement: Supplement 2. — Data Sharing Statement [file jamanetwopen-e2444245-s002.pdf]

Data Sharing Statement

Ma. Perceived Mistreatment and Professional Identity of Medical Students in China. *JAMA Netw Open*. Published November 08, 2024. doi:10.1001/jamanetworkopen.2024.44245

Data
Data available: No

Additional Information
Explanation for why data not available: Quantitative individual participant data from the survey are confidential due to Chinese privacy legislation. Researchers interested in addressing research questions related to this article could be directed to the corresponding author at wuhongbin@pku.edu.cn.
